# Association of Red Blood Cell Distribution Width and Neutrophil-to-Lymphocyte Ratio with Calcification and Cardiovascular Markers in Chronic Kidney Disease

**DOI:** 10.3390/metabo13020303

**Published:** 2023-02-17

**Authors:** Stefanos Roumeliotis, Ioannis E. Neofytou, Cecile Maassen, Petra Lux, Konstantia Kantartzi, Evangelos Papachristou, Leon J. Schurgers, Vassilios Liakopoulos

**Affiliations:** 1 Division of Nephrology and Hypertension, 1st Department of Internal Medicine, AHEPA Hospital, School of Medicine, Aristotle University of Thessaloniki, 54636 Thessaloniki, Greece; 2 Department of Biochemistry, Cardiovascular Research Institute Maastricht, Maastricht University Medical Centre, 6200 MD Maastricht, The Netherlands; 3 Department of Nephrology, Democritus University of Thrace, 68100 Alexandroupolis, Greece; 4 Department of Nephrology and Renal Transplantation, Patras University Hospital, 26504 Patras, Greece

**Keywords:** cardiovascular disease, chronic kidney disease, dephosphorylatyed uncarboxylated Matrix Gla Protein, endothelial dysfunction, inflammation, red blood cell distribution width, Neutrophil-to-lymphocyte ratio, vascular calcification

## Abstract

We aimed to investigate the association between Red Blood Cell Distribution Width (RDW) and Neutrophil-to-Lymphocyte Ratio (NLR), simple, rapidly assessed markers from the complete blood count with vascular calcification (VC)/stiffness and cardiovascular disease (CVD) in chronic kidney disease (CKD). Dephosphorylated, uncarboxylated matrix Gla-protein (dp-ucMGP), and central/peripheral hemodynamics’ parameters were measured in 158 CKD patients, including Hemodialysis and Peritoneal Dialysis. Spearman’s rho analysis showed that RDW correlated with C-reactive protein (CRP) (r = 0.29, *p* < 0.001), dp-ucMGP (r = 0.43, *p* = < 0.0001), central diastolic blood pressure (DBP) (r = −0.19, *p* = 0.02), and albuminuria (r = −0.17, *p* = 0.03). NLR correlated with the duration of CVD (r = 0.32, *p* < 0.001), CRP (r = 0.27, *p* = 0.01), dp-ucMGP (r = 0.43, *p* < 0.0001), central DBP (r = −0.32, *p* < 0.0001) and eGFR (r = −0.25, *p* = 0.04). In multiple regression models, circulating dp-ucMGP was an independent predictor of RDW (β = 0.001, *p* = 0.001) and NLR (β = 0.002, *p* = 0.002). In CKD patients, RDW and NLR are associated with traditional and novel markers of VC and CVD.

## 1. Introduction

Cardiovascular disease (CVD) is highly prevalent, in particular in the early stages of chronic kidney disease (CKD), it progresses in parallel with the deterioration of kidney function and accounts for more than 50% of all deaths in patients with end-stage kidney disease (ESKD) [1]. CVD is the leading cause of death not only in ESKD but also in CKD patients; patients at the early stages of CKD are more likely to die due to CVD or heart failure (HF) than progress to EKSD and require dialysis [2]. This CV burden in uremic patients might be attributed to the fact that arterial calcification and stiffness—which predispose to atherosclerosis and CVD—are gradually increased with the progression of CKD to ESKD and are further exacerbated in dialysis [3]. In CKD, accumulating data suggest that dephosphorylated uncarboxylated matrix Gla-protein (dp-ucMGP), the inactive form of MGP, is a strong and reliable marker of vascular calcification (VC) and predicts CVD morbidity and mortality [4,5,6,7], whereas pulse wave velocity (PWV) has been shown to be a surrogate marker for arterial stiffness in these patients [8].

In order to prevent and manage CV morbidity and mortality, the early and precise identification of patients at risk for arterial calcification or stiffness is crucial in CKD. In this direction, various markers have been proposed; however, the major limitations for the broad clinical use of these markers include high cost and complex, time-consuming methods of measurement. On the other hand, there is a growing interest in the identification of novel, simple, low-cost and easily accessible tools that can be used in everyday clinical practice to classify CKD patients at high risk for VC, vascular stiffness (VS) and CVD.

Red Blood Cell Distribution Width (RDW) is a measure of difference in volume and size of erythrocytes calculated by automated hematology analyzers and routinely reported in the total blood count lab test. RDW is used as a marker of anisocytosis and traditionally has been used for the differential diagnosis of different types of anemias [9]. Increased RDW levels reflect short red blood cell (RBC) life span due to limited erythropoiesis and accelerated RBC destruction. However, large cohort studies during the past decade coherently showed that RDW is steadily increased in CVD [10], heart failure (HF) [11,12,13,14] and CKD [15]. Moreover, it has been reported to be a strong and independent predictor of CV mortality and VC in CKD populations [16,17,18,19].

Although the exact pathophysiology linking increased RDW with CVD has not yet been fully elucidated, VC and VS, chronic inflammation and nutritional disorders have been proposed as potential underlying mechanisms.

The Neutrophil-to-Lymphocyte Ratio (NLR) is a marker also derived from the full blood count lab test, calculated from deriving the absolute number of neutrophils to the absolute count of lymphocytes. Similarly to RDW, NLR has been demonstrated by large studies to be an indicator of CVD, VC and inflammation [20], especially in CKD and ESKD patients [21]. However, until now, no study has assessed the possible clinical value of these two markers combined in CKD cohorts. The aim of this study is to investigate the association of simple, quick and low-cost markers derived from the total blood count, such as RDW and NLR with traditional or non-traditional risk factors of CVD and VC/VS assessed by dp-ucMGP and PWV.

## 2. Materials and Methods

### 2.1. Patients

**Figure 1:**
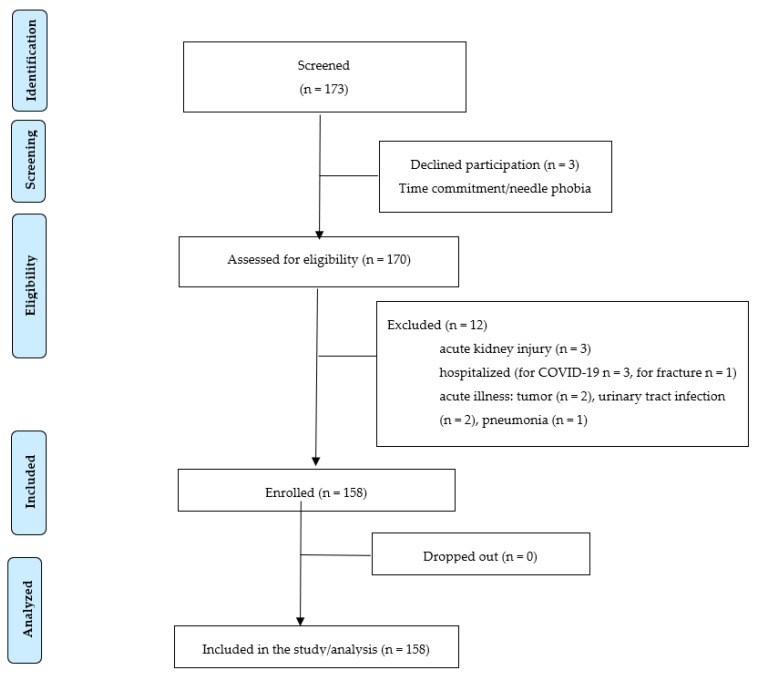
Patients’ enrolment flow chart.

In this cross-sectional, single-center study, we recruited 158 patients at different CKD stages. Eligible patients were those with a documented diagnosis of CKD. A total of 158 patients were included in the study, divided into 73 pre-dialysis outpatients and 84 dialysis patients. All pre-dialysis CKD subjects were outpatients followed in the Division of Nephrology and Hypertension of the University General AHEPA Hospital of Thessaloniki (Greece), whereas dialysis patients were undergoing maintenance hemodialysis in the Hemodialysis Unit or Peritoneal Dialysis, followed in the Peritoneal Dialysis Unit of the University General AHEPA Hospital of Thessaloniki (Greece). All patients were enrolled between 1 November 2021 and 1 March 2022. Initially, 173 patients were screened to participate in the study. Three declined participation and 12 met the exclusion criteria, as described in the patients’ enrolment flow chart (Figure 1).

Among pre-dialysis patients, 11 were at stages 1–2, 2 at stage 3, 33 at stage 4 and 3 at stage 5 (end-stage kidney disease-ESKD), whereas 25 patients were under chronic peritoneal dialysis (PD) and 59 under maintenance hemodialysis (HD) treatment. The estimated glomerular filtration rate was calculated using the CKD-EPI calculation and a diagnosis/classification of CKD was based on the National Kidney Foundation Kidney Disease Outcomes Quality Initiative criteria [22].

Patients with acute kidney injury, hospitalized or acute illness were excluded from the study. The definition of AKI was in accordance with the Acute Kidney Injury Working Group of KDIGO (Kidney Disease: Improving Global Outcomes) [23]. Acute illness was defined as any acute condition that would limit the ability of the patients to participate in the study and might affect the markers measured, such as infection, newly diagnosed tumor and current hospitalization (none of the patients assessed was recently hospitalized, within 3 months from enrollment). At recruitment, we documented demographic, anthropometric, clinical and laboratory data, including documented history of hypertension (HT), type 2 diabetes mellitus (T2DM), heart failure (HF), CVD and measured pulse-wave velocity (PWV), and indices of central blood pressure (BP). CVD included coronary heart disease, heart failure, angina, stroke or peripheral arterial disease. We obtained serum, plasma and whole blood from all patients and urine albumin to creatin ratio (UACR) was measured in a spot urine sample. We measured dp-ucMGP from plasma in 116 patients and obtained RDW and NLR values from all patients from the complete blood count. All patients provided informed consent at enrollment. The study was conducted in accordance with the Helsinki Declaration of Human Rights and was approved by the Ethics Committee/Scientific Council of the Medical School of Aristotle University of Thessaloniki (235/14 May2021).

### 2.2. Laboratory Analyses

We drew fasting blood from all patients into tubes containing EDTA and tubes without anticoagulant and obtained plasma, serum and whole blood. Samples for creatinine, calcium, phosphorus, C-reactive protein (CRP), parathormone, glycated hemoglobin (HbA1c), triglycerides, total, low density lipoprotein (LDL) and high-density lipoprotein (HDL) cholesterol and serum albumin and total blood count for white blood cells, hemoglobin and RDW were transferred to the laboratory and immediately assessed, whereas for dp-ucMGP, we centrifuged the samples and stored plasma at −20 °C, until analysis. We collected blood samples from HD patients after eight hours of fasting overnight and before the start of a mid-week dialysis session. The presence of albuminuria (UACR) was defined in 2 out of 3 consecutive measurements in morning spot urine samples, during a 3-month period.

Plasma dp-ucMGP levels were measured in a single run by the Laboratory of Coagulation Profile (Maastricht, the Netherlands) using the commercially available IVD CE-marked chemiluminescent InaKtif MGP assay on the IDS-iSYS system (IDS, Boldon, UK), which has been described elsewhere [24].

In brief, plasma samples and internal calibrators were incubated using magnetic particles that were coated with murine monoclonal antibodies against dp-MGP, acridinium-labelled murine monoclonal antibodies against ucMGP and an assay buffer. The magnetic particles were captured using a magnet and washed to remove any unbound analyte. Trigger reagents were added, and the resulting light emitted by the acridinium label was directly proportional to the level of dp-ucMGP in the sample. The assay-measuring range was between 300 and 12,000 pmol/L and was linear up to 11,651 pmol/L. The within-run and total variations of this assay were 0.8–6.2% and 3.0–8.2%, respectively. 

RDW and neutrophils/lymphocytes were measured as part of the routine total blood cell count, using the automatic hematology analyzer Sysmex XE-5000 (Sysmex Corporation, Kobe, Japan). According to our laboratory, the reference range for RDW was 12.0–14.0%. 

To assess the potential clinical utility of RDW and NLR, all patients were divided into 2 groups according to the presence of CVD and/or HF and into quartiles according to median RDW and NLR.

### 2.3. PWV, Peripheral and Central Hemodynamic Parameters Measurement

Peripheral (brachial) and central aortic BP, diastolic (DBP), systolic (SBP), pulse wave velocity (PWV), cardiac rhythm, pulse pressure and augmentation index (AI) and other peripheral and central hemodynamic parameters were determined with the Mobil-O-Graph device (IEM, Stolberg, Germany), as described before [25]. The BP-detection unit was validated according to the ESH/ESC criteria, as described elsewhere [26].

### 2.4. Statistics

We performed all statistical analyses using the IBM Statistical Package for Social Sciences (SPSS 18.0 for Windows, IBM, Chicago, IL, USA). The Kolmogorov–Smirnov test was used to test data for normality. Normally distributed continuous variables are presented as mean ± standard deviation and non-normally distributed continuous variables are presented as median (range, minimum to maximum values). Patient characteristics were compared among groups of HF/CVD diagnosis and RDW/NLR quartiles using the chi-square test for categorical variables and the Mann–Whitney test for continuous variables. To compare the values of NLR among groups with different cause of nephropathy, the Kruskal–Wallis test was used. Bivariate associations between RDW, NLR and other variables were examined with Spearman’s correlation co-efficient. We divided our cohort into quartiles according to median RDW (14.4%) and median NLR (3.3)—quartile 1: below median RDW below median NLR; quartile 2: below median NLR, above median RDW; quartile 3: above median NLR, below median RDW and quartile 4: above median RDW, above median NLR. The Kruskal–Wallis test or chi-square test was used to compare for differences of variables among groups, accordingly. To investigate the possible predictors of RDW and NLR, we conducted a multiple regression analysis (forward, stepwise) with RDW and NLR as the dependent variables in the absence and presence of possible predictors, as determined in the correlation bivariate analyses. The multiple regression models where RDW was the dependent variable were adjusted for serum albumin, C-reactive protein, urinary albumin–creatinine ratio, calcium, phosphorus, cardiac rhythm, central diastolic blood pressure, warfarin and erythropoietin-stimulating agent, whereas the models with NLR as the dependent variable were adjusted for age, presence of cardiovascular disease, duration of cardiovascular disease, serum albumin, C-reactive protein, estimated glomerular filtration rate, calcium, parathormone, central diastolic blood pressure, cause of nephropathy and erythropoietin-stimulating agent. Non-normally distributed variables were log-transformed before entering the regression analyses. Significance was set at *p* < 0.05.

## 3. Results

In the study cohort, 41 patients had HF (25.9%), 61 CVD (38.6%) and 81 (51.3%) had HF and/or CVD. Clinical, anthropometric, hematologic and biochemical characteristics of 158 CKD patients according to the presence of HF/CVD are shown in Table 1. Patients with a documented history of HF or CVD were mostly male, had significantly higher RDW, NLR and dp-ucMGP, were older and had significantly lower eGFR and HDL cholesterol. They also had a 28.3% increased prevalence of T2DM diagnosis, longer period of T2DM diagnosis and significantly higher HbA1c values. Compared to patients with no HF or CVD, those with HF and/or CVD presented significantly lower diastolic, central diastolic and mean BP. There was no difference among groups regarding BMI, SBP, PWV, AI, calcium, phosphorus, parathormone, CRP, UACR or treatment with warfarin or erythropoietin. In the subgroup of 84 ESKD patients undergoing maintenance dialysis (25 PD and 59 HD), time on dialysis, Kt/V (indicator of dialysis efficiency) and peritoneal equilibration test and type of PD transporter did not differ significantly among groups of HF or CVD (results not shown). Moreover, the prevalence of HF/CVD did not differ significantly among PD and HD (56% in PD and 52.5% in HD).

The correlation matrix analysis of RDW and NLR with various traditional and non-traditional risk factors (categorized as anthropometric, hematologic, nutrition, inflammation, CKD and calcification/stiffness markers) for HF and CVD in CKD is shown in Table 2. RDW was positively correlated with NLR (*r* = 0.23, *p* = 0.004), total WBC (*r* = 0.17, *p* = 0.04), CRP (*r* = 0.29, *p* < 0.001), phosphorus (*r* = 0.27, *p* = 0.001) and dp-ucMGP (*r* = 0.43, *p* = < 0.0001). There was a significant inverse correlation between RDW and hemoglobin (*r* = −0.36, *p* < 0.0001), DBP (*r* = −0.19, *p* = 0.02), central DBP (*r* = −0.19, *p* = 0.02), cardiac rhythm (*r* = −0.16, *p* = 0.05), serum albumin (*r* = −0.24, *p* = 0.002), UACR (*r* = −0.17, *p* = 0.03) and calcium (*r* = −0.23, *p* = 0.004). NLR was positively correlated with total WBC (*r* = 0.34, *p* < 0.001), age (*r* = 0.19, *p* = 0.02), duration of CVD (*r* = 0.32, *p* < 0.001), CRP (*r* = 0.27, *p* = 0.01), dp-ucMGP (*r* = 0.43, *p* < 0.0001) and parathormone (*r* = 0.18, *p* = 0.024) and negatively with DBP (*r* = −0.30, *p* < 0.0001), central DBP (*r* = −0.32, *p* < 0.0001), mean BP (*r* = −0.24, *p* = 0.003), hemoglobin (*r* = −0.37, *p* < 0.0001), serum albumin (*r* = −0.29, *p* < 0.0001), calcium (*r* = −0.23, *p* = 0.005) and eGFR (*r* = −0.25, *p* = 0.04).

Moreover, the Kruskal–Wallis analysis showed that NLR was significantly different among groups with a different cause of nephropathy (*p* = 0.017), whereas men presented significantly higher values of NLR compared to women (3.56 vs. 2.78, *p* = 0.008). Patients receiving an erythropoietin-stimulating agent and those with documented CVD had significantly higher values of NLR compared to the others (*p* < 0.0001 and *p* < 0.0001, respectively). In the group analysis, RDW was associated with use of warfarin and use of erythropoietin; patients treated with warfarin had significantly higher values of RDW compared to those who did not receive warfarin (17.1 vs. 14.4, *p* = 0.006). Similarly, erythropoietin was associated with an increase of 1.3% in RDW (*p* < 0.0001).

**Figure 2:**
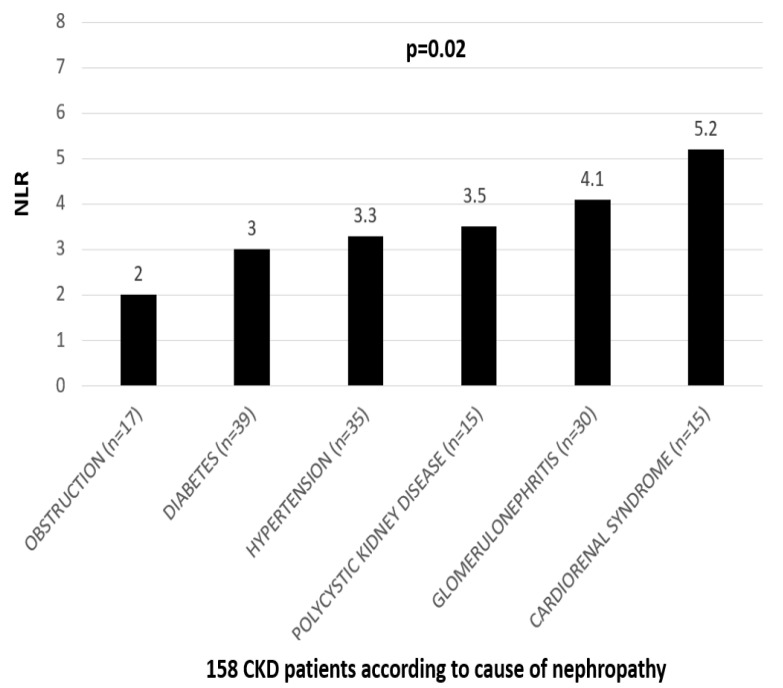
NLR according to different causes of nephropathy in the cohort of 158 CKD patients.

NLR values according to different causes of nephropathy are shown in Figure 2. Patients with obstructive nephropathy had lower NLR values and patients with cardiorenal syndrome, due to heart failure with reduced ejection fraction, had the highest (2 and 5.2, respectively). Compared to patients with diabetic, hypertensive and polycystic nephropathy, those with glomerulopathy had significantly higher NLR values (3, 3.3, 3.5 and 4.1, respectively). Therefore, NLR was significantly higher in patients with CVD-derived CKD than those with glomerulopathy, where inflammation is a major pathogenetic mechanism.

We divided our cohort to quartiles according to median RDW (14.4%) and median NLR (3.3)—quartile 1: below median RDW below median NLR; quartile 2: below median NLR, above median RDW; quartile 3: above median NLR, below median RDW and quartile 4: above median RDW, above median NLR (Table 3). Dp-ucMGP and the prevalence of CVD were progressively increased across quartiles. Compared to the first quartile, patients at the fourth quartile had significantly lower Hb, DBP, mean BP, central DBP, calcium and serum albumin values and higher duration of CVD, phosphorus and CRP levels.

Table 4 shows the multiple regression analysis in the study cohort with RDW as the dependent variable. In both unadjusted and adjusted for several well-established confounders affecting RDW values (serum albumin, CRP, UACR, calcium, phosphorus, cardiac rhythm, central DBP, warfarin, ESA), only circulating dp-ucMGP was an independent predictor of RDW (β = 0.001, *p* = 0.001).

Table 5 shows the multiple regression analysis in the study cohort with NLR as the dependent variable. Even after adjustment for all variables correlated with NLR in the previous analyses (age, CVD, duration of CVD, serum albumin, CRP, eGFR, calcium, parathormone, central DBP, cause of nephropathy, ESA), dp-ucMGP, mean BP and gender were strong, independent predictors of NLR.

## 4. Discussion

CVD still remains the leading cause of morbidity and mortality in CKD patients, accounting for more than 50% of all deaths in ESKD. VC is highly prevalent in CKD patients and might explain the heavy CV burden that these patients carry. Therefore, the investigation of early, accurate biomarkers that might identify and classify CKD patients at risk for VC/VS is mandatory. However, although several markers have shown promising results, their use in everyday clinical practice is discouraged due to several limitations including high cost and/or complex or time-consuming measurement.

We found that CVD was highly prevalent in our study cohort (38.6%) and associated with RDW, NLR and dp-ucMGP. Further, when we divided our cohort into quartiles according to median values of RDW and NLR, we found that compared to the lowest quartile, patients with values above median of both RWD and NLR had significantly higher dp-ucMGP, prevalence of CVD, longer duration of T2DM, lower diastolic/mean and central diastolic BP, lower calcium and albumin and higher CRP and phosphorus, known traditional and non-traditional CV risk factors. Moreover, RDW was positively correlated with NLR, CRP, dp-ucMGP and phosphorus and negatively with peripheral and central DBP, UACR, calcium and albumin, whereas NLR was positively correlated with age, duration of CVD, CRP, PTH, dp-ucMGP, and inversely with peripheral and central DBP, calcium and albumin.

RDW is a marker measuring the difference/variation in the size and volume of erythrocytes routinely reported in the total blood count. It is calculated from the division of the standard deviation of the mean corpuscular volume (MCV) by the MCV multiplied by 100 to yield a percentage value. The normal range of RDW is roughly between 11.5 and 15.4%. RDW was long used for the differential diagnosis of anemias; however, in recent years, it became evident that it might serve as a novel predictive marker of CVD and mortality in various settings, including the general population [27], HF [14,28,29] and CVD [30,31,32].

In line with these, a meta-analysis including 28 studies and approximately 103,000 patients with CVD showed that RDW was an independent predictor of adverse events including major CV events [33]. However, in CKD populations, the data regarding the association of RDW with CVD and mortality are mainly focused in ESKD populations, either HD [34,35] or PD [36,37,38]. In these patients, even a modest 1% increase in RDW levels increases the risk for all-cause mortality by 47% [39]. In pre-dialysis CKD, the data remain limited and are mainly derived from three retrospective studies [16,17,18,40]. In agreement with our results, we have previously showed in a cohort of 142 diabetic CKD patients in various CKD stages that RDW was an independent predictor of CV disease and mortality [17]. This association might be explained by the fact that VC (assessed by carotid intima media thickness) was the strongest independent factor predicting RDW values. In agreement with these findings, we found that dp-ucMGP (a well-established marker of VC) was independently associated with RDW values. Although VC was long considered a passive, degenerative process of the chronic accumulation of calcium in the arterial wall, this perspective recently changed and it is now well accepted that the calcification process is an active process that is regulated by molecules and proteins that act either as inhibitors or promoters [5,41]. Among these, MGP, a vitamin K-dependent protein, is the strongest natural inhibitor of VC that might abbrogate or even reverse the calcification process. To become active, MGP must undergo carboxylation and phosphorylation. MGP carboxylation requires vitamin K to be a cofactor to drive this reaction [42]. The fully inactive form of MGP, dp-ucMGP, reflects vitamin K deficiency and has been shown to be a reliable marker of arterial calcification and stiffness in various settings, including uremia. In CKD, dp-ucMGP is progressively increased in parallel with CKD stages [4,7], is tightly correlated with various surrogate markers of VC or VS (including cIMT, PWV, coronary calcification, Agatston score, etc.) and has been repeatedly shown to be a strong independent predictor of CVD [6,43]. Therefore, since vitamin K deficiency (assessed by increased dp-ucMGP) has been coherently shown to be a novel, non-traditional risk factor for CVD in CKD, several ongoing randomized controlled studies are currently investigating whether exogenous supplementation with vitamin K might ameliorate VC and protect from CVD by reducing circulating dp-ucMGP in CKD, HD and PD patients [42,44,45].

In our study, we found that both RDW and NLR were strongly associated with circulating dp-ucMGP; moreover, dp-ucMGP was the strongest independent factor predicting the values of these two markers, thus indicating that both RDW and NLR are associated with accelerated VC and an increased risk for CVD. According to our knowledge, this is the first study to report an association between RDW, NLR and dp-ucMGP. However, RDW has been repeatedly associated with other surrogate calcification markers, such as IMT [17,19,46,47,48], coronary calcium scores [49] and PWV [50,51]. 

In disagreement with Fornal et al. [52], we failed to show any association between RDW and PWV. However, this study included only hypertensive patients. Similar results were reported by two other small cohort studies in kidney transplant recipients [50,51]. One possible explanation might be the different population we enrolled and the fact that we used the optimal oscillometric method for measuring PWV, whereas the tonometric method is more sensitive and accurate [53]. Nonetheless, some indices of PWV (central diastolic blood pressure, diastolic blood pressure and heart rate) were found to be significantly correlated with RDW and NLR. Moreover, we found that RDW and NLR were associated with calcium, phosphate and PTH well-established traditional factors favoring VC in HD patients [54,55,56]. The associations of RDW with markers of VC and CVD might be attributed to shortened RBC life span, inhibited erythropoietin response, anemia and impaired iron metabolism. However, the correlation of RDW with VC was independent of Hb levels and the use of ESAs. Moreover, we found that patients receiving warfarin (a vitamin K antagonist) had significantly higher RDW levels, thus indicating that RDW is directly associated with vitamin K deficiency. Another explanation for our findings might be that anisocytosis might reflect a high inflammatory state and progressive CKD (as assessed by high UACR). The association between RDW, albuminuria [17,57,58] and inflammation in CKD settings [17,19,35,59] has been repeatedly reported before.

We also found that besides RDW, NLR was associated with CRP, dp-ucMGP, CVD duration, indices of peripheral and central hemodynamics and eGFR. Although several biomarkers have been proposed as markers of VC and CVD in CKD, most of them necessitate special laboratory equipment or assays, need time-consuming methods or are expensive. In contrast, both RDW and NLR are simple, low in cost and quickly extracted by the complete blood count. NLR in peripheral blood is calculated by dividing the absolute number of neutrophils by the absolute number of lymphocytes, with data obtained by the total blood count. In healthy subjects, the normal NLR values are approximately between 0.8 and 3, whereas higher values indicate stress; in critically ill patients, NLR values might even reach 30 times higher than normal range values. NLR is a surrogate and reliable marker of systemic inflammation and has emerged during the past decade as a predictive biomarker for mortality and CVD in various populations, especially in CKD patients [21,60,61]. In our study, we found a tight correlation between NLR and CVD duration and traditional/non-traditional markers of CVD/VC. In agreement with our results, a very recent meta-analysis including 13 studies and 116,709 CKD patients showed that high NLR was a significant, strong predictor of both all-cause and CVD mortality (HR 1.93, 95% CI 1.87–2.00; *p* < 0.00001 and HR 1.45, 95% CI 1.18–1.79, *p* < 0.001, respectively) [62]. The association between NLR and VC is scarcely documented in CKD patients. Wang et al. reported that NLR was associated with coronary artery calcification in CKD stage 3–5 patients [63], whereas Li et al. showed that NLR was an independent risk factor of cardiac valve calcification in CKD patients [64] and another three small studies showed that VC was correlated with NLR in dialysis populations [65,66,67]. In our study, we found a strong association between NLR and the disturbed homeostasis of calcium–phosphate–PTH, which is a well-known molecular pathway underlying VC in uremic patients [68]. Moreover, we found that high NLR was correlated with peripheral and central hemodynamic parameters obtained by PWV measurements, which is in agreement with a previous study showing a close association between NLR and arterial stiffness in PD patients [69]. However, the main finding of our study was that dp-ucMGP, a reliable and novel marker of VC and vitamin K deficiency, was the strongest independent predictor of NLR values. According to our knowledge, this is the first study to ever report this association. We found that NLR was associated with eGFR and the presence of cardiorenal syndrome and/or glomerulopathy in our cohort. This finding could be interpreted by the fact that NLR is an independent risk factor for both CKD and HF/CVD [70,71]. Glomerulonephritis, on the other hand, is primarily an inflammatory disease which results in a congregation of inflammatory molecules that damages the glomeruli. Likewise, several investigators have previously reported that NLR was not only associated with renal function [72,73,74] but also predicted the deterioration of kidney function and CVD [21,75,76,77] in various types of glomerulopathies including lupus, vasculitis and IgA nephropathy. 

Our findings that both RDW and NLR were associated with VC, CVD and kidney function in a cohort of CKD patients might be explained by the fact that these two markers reflect systemic inflammation [78,79,80]. Since we found associations with both VC and VS, it could be hypothesized that both markers might reflect endothelial dysfunction in these patients. 

In this study, we found that parameters from the total blood count (RDW and NLR) are associated with inflammation, markers of CKD and traditional/non-traditional markers of endothelial dysfunction in a cohort of 158 CKD patients. Moreover, we found that circulating dp-ucMGP was the strongest predictor of both NLR and RDW. According to our knowledge, this is the first study to investigate the association between RDW, NLR and dp-ucMGP and other markers of VC or stiffness in a cohort of CKD patients in different stages. The strengths of our study include the large sample size, comprehensive data and the inclusion of several parameters to improve the statistical validity of our results. However, there are various limitations that should be acknowledged. Firstly, due to the cross-sectional, observational design, no causality can be established, and our results should be interpreted with caution. However, based on the results we obtained, we started following this cohort prospectively to investigate the possible predictive effect of the markers tested for CV events, mortality and CV mortality. Secondly, we cannot draw any definite conclusions regarding the mechanisms underlying the associations that were found, and thirdly, the single center design and the fact that other important parameters-markers of VC/VS were not included in our study. However, our study showed that information derived from a very simple, cheap and quick exam, the full blood count, might give a rough idea regarding the status of VC and/or CVD in uremic patients that carry a heavy CV burden. Therefore, our conclusions are speculative and require further, larger, prospective cohort studies in order to draw definite conclusions regarding the possible clinical utility of RDW and NLR.

## Figures and Tables

**Table 1:** Clinical, anthropometric and biochemical characteristics of chronic kidney disease patients with and without HF/CVD. Results for continuous variables are presented as median (range) or mean (S.D.).

	All Patients (*n* = 158)	HF/CVD (*n* = 81)	No HF no CVD (*n* = 77)	*p*
RDW (%)	14.4 (11.4–39.7)	14.6 (11.7–28.0)	14.3 (11.4–39.7)	0.05
Hemoglobin (g/dL)	12.0 ± 1.7	11.8 ± 1.8	12.3 ± 1.6	0.022
WBC (10^9^/L)	7.86 (2.17–16.72)	8.235 (3.76–16.72)	7.40 (2.17–14.67)	0.027
NEUT (10^9^/L)	5.30 (1.0–15.6)	5.69 (2.25–15.6)	4.7 (1.0–10.5)	0.005
LYMPH (10^9^/L)	1.45 (0.49–4.09)	1.285 (0.49–4.09)	1.59 (0.64–3.95)	0.042
NLR	3.3 (0.7–23.3)	3.7 (1.3–23.3)	2.8 (0.7–9.0)	0.001
Dp-ucMGP (pM)	958 (298–5470)	1024 (351–5000)	918 (298–5470)	0.009
Age (years)	68.5 (25–89)	71 (33–89)	62 (25–89)	<0.0001
Gender, Male (%)	69.0	77.8	59.7	0.011
Hypertension (yes,%)	76.6	74.0	79.2	0.28
Duration of HT (years)	11.5 ±10.3	12.6 ±11.1	10.3 ±9.3	0.25
T2DM (yes,%)	40.5	54.3	26.0	<0.0001
Duration of T2DM (years)	5.7 ± 8.5	7.9 ± 9.3	3.4 ± 7.0	<0.0001
BMI (kg/m^2^)	26.4 (18.9–43.8)	26.9 (19–43.8)	26.1 (18.9–42.2)	0.15
SBP (mm Hg)	134.1 ± 22.1	131.9 ± 23.8	136.4 ± 20.0	0.19
DBP (mm Hg)	82.1 ± 13.1	79.5 ± 13.1	84.8 ± 12.6	0.005
Mean BP (mm Hg)	106.0 ± 15.8	103.5 ± 16.6	108.6 ± 14.7	0.03
Peripheral pulse pressure	48 (16–97)	48 (16–97)	48 (27–92)	0.93
Cardiac Rythm	74.6 ± 13.5	72.6 ± 11.6	76.8 ± 15.0	0.09
Central SBP (mm Hg)	122.3 ± 20.3	119.8 ± 21.3	125.0 ± 19.0	0.09
Central DBP (mm Hg)	83.7 ± 13.2	80.7 ± 13.4	86.7 ± 12.3	0.002
Central pulse pressure	36 (12–79)	37 (12–77)	36 (18–79)	0.78
AI	23.3 ± 10.4	23.3 ± 10.4	23.3 ± 10.5	0.14
PWV	8.2 (5.8–12.1)	8.7 (6.2–12.1)	8 (5.8–12.0)	0.24
EGFR (ml/min/1.73m^2^)	36.4 ± 20.2	31 ± 15	41.5 ± 23.2	0.05
Calcium (mg/dL)	8.9 ± 0.8	8.9 ± 0.7	8.9 ± 0.8	0.79
Phosphorus (mg/dL)	4.2 (2.3–11)	4.2 (2.4–11)	4.2 (2.3–8.4)	0.89
Parathormone (pg/mL)	131 (7.2–892)	120 (10–892)	139 (7.2–820)	0.76
Albumin (g/dL)	4.1 (2.4–6.8)	4.1 (2.4–5.0)	4.1 (2.6–6.8)	0.18
Total cholesterol (mg/dL)	156.4 ± 44.0	153.9 ± 43.5	159.0 ± 44.8	0.52
LDL-cholesterol (mg/dL)	81.7 ± 38.0	80.4 ± 37.8	83.0 ± 38.3	0.73
HDL-cholesterol (mg/dL)	44.3 ± 17.7	40.6 ± 13.5	48.2 ±20.6	0.02
Triglycerides (mg/dl)	138 (17–402)	140 (17–402)	134 (48–342)	0.79
HbA1c (%)	5.6 (3–10.7)	5.9 (4.2–9.6)	5.3 (3–10.7)	<0.0001
CRP (mg/dL)	0.52 (0.1–77.0)	0.52 (4.2–9.6)	0.50 (0.1–77)	0.27
UACR (mg/g)	30 (0–5800)	30 (0–2900)	20 (0–5800)	0.64
Warfarin treatment (yes, n)	8	6	2	0.16
Treatment with ESA (yes, n)	76	41	35	0.31

*p* values of the Mann–Whitney test or the chi-square test for differences of variables among groups. RDW, Red Blood Cell Distribution Width; WBC, White Blood Cells; NEUT, Neutrophils, LYMPH, Lymphocytes; NLR, Neutrophil-to-Lymphocyte Ratio; Dp-ucMGP, Dephosphorylated Uncarboxylated Matrix Gla Protein; HT, Hypertension; T2DM, Type 2 Diabetes Mellitus; BMI, Body Mass Index; SBP, Systolic Blood Pressure; DBP, diastolic Blood Pressure; BP, Blood Pressure; AI, Augmentation Index; PWV, Pulse Wave Velocity; LDL, low-density lipoprotein; HDL, high-density lipoprotein; HbA1c, glycated hemoglobin A1c; CRP, C-reactive protein; eGFR, estimated glomerular filtration rate; UACR, urinary albumin–creatinine ratio; ESA, erythropoietin-stimulating agent.

**Table 2:** Correlations of various parameters with RDW and NLR.

	RDW			NLR
Parameters	*r*	*p*	*r*	*p*
Anthropometric parameters				
Age (years)	0.12	0.15	0.19	0.02
HT duration (years)	0.05	0.52	0.08	0.31
T2DM duration (years)	−0.05	0.96	0.04	0.67
CVD duration (years)	0.15	0.07	0.32	<0.0001
SBP (mm Hg)	−0.10	0.23	−0.14	0.08
DBP (mm Hg)	−0.19	0.02	−0.3	<0.0001
Mean BP (mm Hg)	−0.16	0.052	−0.24	0.003
BMI (kg/m^2^)	−0.01	0.89	−0.04	0.61
Hematologic parameters from total blood count				
RDW (%)	-	-	0.23	0.004
NLR	0.23	0.004	-	-
Hemoglobin (g/dL)	−0.36	<0.0001	−0.37	<0.0001
WBC (10^9^/L)	0.17	0.04	0.34	<0.0001
Nutrition–inflammation markers				
Albumin (g/dL)	−0.24	0.002	−0.29	<0.0001
Total chol (mg/dL)	−0.15	0.06	0.008	0.92
LDL chol (mg/dL)	−0.08	0.33	0.09	0.25
HDL chol (mg/dL)	−0.14	0.09	−0.07	0.40
Triglycerides (mg/dL)	0.02	0.85	0.05	0.51
HbA1c (%)	−0.04	0.61	0.05	0.57
CRP (mg/dL)	0.29	<0.001	0.27	0.01
CKD markers				
eGFR (mL/min/1.73 m^2^)	−0.22	0.08	−0.25	0.04
UACR (mg/g)	−0.17	0.03	−0.06	0.47
Markers of vascular dysfunction				
Dp-ucMGP (pM)	0.43	<0.0001	0.43	<0.0001
Calcium (mg/dL)	−0.23	0.004	−0.23	0.005
Phosphorus (mg/dL)	0.27	0.001	0.11	0.17
Parathormone (pg/mL)	0.14	0.10	0.18	0.024
PWV	−0.01	0.94	−0.05	0.57
Peripheral pulse pressure	0.01	0.87	0.02	0.86
Cardiac rhythm (bpm)	−0.16	0.05	−0.14	0.08
Central SBP (mm Hg)	−0.10	0.23	−0.15	0.06
Central DBP (mm Hg)	−0.19	0.02	−0.32	<0.0001
Central pulse pressure	0.03	0.72	0.07	0.42
AI	0.13	0.11	0.05	0.56

Spearman’s rho test correlation. RDW, Red Blood Cell Distribution Width; WBC, White Blood Cells; NEUT, Neutrophils, LYMPH, Lymphocytes; NLR, Neutrophil-to-Lymphocyte Ratio; Dp-ucMGP, Dephosphorylated Uncarboxylated Matrix Gla Protein; HT, Hypertension; T2DM, Type 2 Diabetes Mellitus; BMI, Body Mass Index; SBP, Systolic Blood Pressure; DBP, Diastolic Blood Pressure; BP, Blood Pressure; AI, Augmentation Index; PWV, Pulse Wave Velocity; LDL, low-density lipoprotein; HDL, high-density lipoprotein; HbA1c, glycated hemoglobin A1c; CRP, C-reactive protein; eGFR, estimated glomerular filtration rate; UACR, urinary albumin–creatinine ratio.

**Table 3:** Clinical, anthropometric and biochemical characteristics of chronic kidney disease patients according to quartiles of NLR and RDW. Results for continuous variables are presented as median (range) or mean (S.D.).

	Quartile 1 NLR ≤ 3.3 and RDW ≤ 14.4 (n = 45)	Quartile 2 NLR ≤ 3.3 and RDW > 14.4 (n = 36)	Quartile 3 NLR > 3.3 and RDW ≤ 14.4 (n = 32)	Quartile 4 NLR > 3.3 and RDW > 14.4 (n = 45)	*p*
RDW (%)	13.3 (11.4–14.3)	15.8 (14.4–39.7)	13.4 (12.3–14.2)	16.0 (14.4–27.9)	<0.0001
NLR	2.1 (0.7–3.3)	2.2 (1.2–6.9)	5.0 (3.3–14.3)	5.6 (3.4–23.3)	<0.0001
Hemoglobin (g/dL)	12.8 ± 1.4	12.0 ± 1.7	12.1 ± 1.9	11.1 ± 1.2	<0.0001
Dp-ucMGP (pM)	800.5 (298–4814)	934 (684–5470)	1015 (566–4090)	1442 (658–5000)	<0.0001
CVD (yes, %)	26.7%	27.7%	44.8%	57.8%	0.008
Duration of CVD (years)	2.7 ±5.9	2.1 ±4.6	3.3 ±4.3	4.9 ±6.4	0.02
DBP (mm Hg)	84.9 ± 11.5	84.2 ± 12.0	83.6 ± 11.9	75.4 ± 12.5	0.001
Mean BP (mm Hg)	108.6 ± 14.7	108.1 ± 14.8	107.2 ± 16.5	99.6 ± 15.0	0.022
Central DBP (mm Hg)	86.6 ± 11.1	86.4 ± 11.6	85 ± 12.0	76.5 ± 12.7	<0.0001
Calcium (mg/dL)	9.2 ± 0.6	9.0 ± 0.8	9.0 ± 0.7	8.6 ± 0.8	0.001
Phosphorus (mg/dL)	3.7 (2.4–6.5)	4.5 (2.5–9.4)	4.1 (2.7–11.0)	4.5 (2.3–7.6)	0.01
Albumin (g/dL)	4.2 (3.4–6.8)	4.1 (3.2–4.9)	4.0 (2.9–5.0)	3.9 (2.4–4.5)	<0.0001
CRP (mg/dL)	0.3 (0.1–15.0)	0.6 (0.1–77.0)	0.5 (0.1–7.7)	0.8 (0.2–23.3)	<0.0001
UACR (mg/g)	263 ±614	198 ±550	550 ±1220	191 ±0.612	0.04
Use of ESA	37.7%	50%	31%	71.1%	0.002

*p* values of the Kruskal–Wallis test or the chi-square test for differences of variables among groups. RDW, Red Blood Cell Distribution Width; NLR, Neutrophil-to-Lymphocyte Ratio; Dp-ucMGP, Dephosphorylated Uncarboxylated Matrix Gla Protein; DBP, Diastolic Blood Pressure; BP, Blood Pressure; CRP, C-reactive protein; UACR, urinary albumin–creatinine ratio; ESA, erythropoietin-stimulating agent.

**Table 4:** Multiple regression analysis (stepwise forward) in 158 CKD patients with RDW as the dependent variable.

	β	SE	*p*	CI
Model 1 (Unadjusted)				
Dp-ucMGP	0.001	<0.0001	0.001	0.00–0.002
Model 2 (Adjusted)				
Dp-ucMGP	0.001	<0.0001	0.001	0.00–0.002

Adjusted for serum albumin, C-reactive protein, urinary albumin–creatinine ratio, calcium, phosphorus, cardiac rhythm, central diastolic blood pressure, warfarin, erythropoietin-stimulating agent. SE, standard error; CI, confidence interval.

**Table 5:** Multiple regression analysis (stepwise forward) in 158 CKD patients with NLR as the dependent variable.

	β	SE	*p*	CI
Model 1 (Unadjusted)				
Dp-ucMGP	0.002	0.001	0.002	0.001–0.003
Model 2 (Adjusted)				
Dp-ucMGP	0.002	<0.001	0.002	0.001–0.003
Mean BP	−0.059	0.019	0.003	−0.097 to −0.021
Sex	1.70	0.60	0.006	0.50–2.90

Adjusted for age, presence of cardiovascular disease, duration of cardiovascular disease, serum albumin, C-reactive protein, estimated glomerular filtration rate, calcium, parathormone, central diastolic blood pressure, cause of nephropathy, erythropoietin-stimulating agent. SE, standard error; CI, confidence interval.

## Data Availability

Data is contained within the article.
